# Mechanisms of Action of Antipsychotic Drugs of Different Classes, Refractoriness to Therapeutic Effects of Classical Neuroleptics, and Individual Variation in Sensitivity to their Actions: PART II

**DOI:** 10.2174/157015909790031184

**Published:** 2009-12

**Authors:** R Miller

**Affiliations:** Otago Centre for Theoretical Studies in Psychiatry and Neuroscience (OCTSPAN), Department of Anatomy and Structural Biology, School of Medical Sciences, University of Otago, P.O.Box 913, Dunedin, New Zealand

**Keywords:** Antipsychotic drugs, neuroleptic drugs, cholinergic interneurones, D1 receptors, D2 receptors, muscarinic M1 receptors, muscarinic M4 receptors, neuroleptic threshold, individualized dose, atypical antipsychotic agents.

## Abstract

Rapid-onset psychotic rebound is uncommon on discontinuation of most antipsychotic drugs, as might be expected for antipsychotic drugs with (hypothetically) indirect actions at their final target receptors. Rapid-onset psychosis is more common on withdrawal of clozapine, which might be expected if its action is direct. Drugs other than clozapine (notably thioridazine) may have hitherto unrecognised similarities to clozapine (but without danger of agranulocytosis), and may be useful in treatment of refractory psychosis. Quetiapine fulfils only some criteria for a clozapine-like drug. Clinical response to neuroleptics varies widely at any given plasma level. Haase’s “neuroleptic threshold” concept suggests that the dose producing the slightest motor side effects produces most or all of the therapeutic benefit, but analyses presented here suggest that antipsychotic actions are not subject to a sharp “all-or-none” threshold but increase over a small dose range. This concept could provide a method for quantitative determination of individualized optimal doses.

## 1. WITHDRAWAL-EMERGENT PSYCHOSIS: EVIDENCE AND THEORY

What happens when antipsychotic drugs are withdrawn, or the dose reduced? This question is not fully resolved. Empirically, for many cases previously receiving standard neuroleptic drugs, it is documented that, if relapse occurs, it does not do so immediately, but in a probabilistic manner, sometime in the next one or two years (see PART I, Sect. 5). The cumulative rate of relapse over this period may vary, according to clinical state at the time of withdrawal [50], in-patient versus out-patient status of the subjects, whether the change is sudden or gradual [123], and in relation to life events [5]. In a second pattern, documented as “supersensitivity psychosis” relapse may occur more rapidly; and, as summarised in PART I (Sect 7), may be the consequence of progressive reduction in the number of striatal cholinergic interneurones in some patients, during prolonged treatment with neuroleptic drugs. That this is not due to reversible changes in receptor numbers is indicated by the relative permanence of the condition, once it has appeared [14,15].

In the case of clozapine there is evidence of a third pattern of events, indicative of direct pharmacodynamic rather than more complex processes at the psychological level: Developing within days of the last dose of clozapine there may be a sudden “rebound”, which may include agitation, abnormal movements, and florid psychosis, often worse than in the original psychotic illness [3,31,113]. This may occur in at least 50% of patients after sudden discontinuation [107]. If clozapine is re-introduced to check the rapid worsening in condition, the dose needed is substantially larger than when initially prescribed [90]. Patients receiving drugs with anti-cholinergic potency (including both antidepressants and standard antipsychotic drugs), in addition to clozapine are less likely to experience rebound psychosis than those not so “protected” (21% *vs* 71% of patients, in a retrospective Finnish study [24,107]). The incidence of such rebound psychosis is much lower after short (28 day) courses of clozapine [108], than in other studies where clozapine was used for longer periods. The rebound psychosis, if it occurs, is transient, lasting about one month, with improvement thereafter [107]. In advanced Parkinson’s disease patients, sudden withdrawal of clozapine may lead to severe exacerbation of the original parkinsonian symptoms [133], and clozapine is not easily replaced by other atypical drugs, such as risperidone or olanzapine [40]. In Western countries clozapine is now generally reserved for refractory patients, in whom this sudden rebound psychosis might be attributed to the resurgence of the original illness. However, when clozapine is given for some time to patients known to be neuroleptic-responsive, its withdrawal is even then accompanied by quick-onset rebound psychosis [84]. Such reactions after clozapine withdrawal are actually more common in patients previously responsive to neuroleptics than in refractory patients [82]. If the withdrawal is achieved by “cross-tapering” with typical drugs, psychotic relapse after the last dose of clozapine is given may still occur also within a few days, despite the expected protection by the standard drug. Replacement by the atypical drug risperidone (which has little cholinergic potency) has little protective effect [122], although this transition is possible, if careful cross-tapering is carried out [42]. If, in the aftermath of withdrawal, such patients are given a typical neuroleptic drug, motor side effects of the latter may be more intense for a while than ever experienced previously with such drugs [8,27,84].

Apart from the pattern described by Chouinard and colleagues, how is one to understand the two other patterns of events, after withdrawal, respectively of standard neuroleptic drugs, and of clozapine? For clozapine withdrawal, the evidence provides many hints of compensatory change in receptor numbers (sub- or supersensitivity), leading transiently to severe pathology. Which are the relevant receptors? Why is this pattern not seen with typical neuroleptics, or other atypical drugs?

Fig. (**1**) is a schematic depiction of events occurring with standard neuroleptic drugs (left), and for clozapine (right). For each class of drug, there are three phases (columns), initial acute treatment, chronic treatment (months or years), and then sudden withdrawal. Six variables (rows) are represented: (i) the number of D2 receptors (located on cholinergic interneurones); (ii) the release of ACh from these neurones (increased, when D2 receptors are blocked by standard antipsychotic drugs); (iii) the number of muscarinic M4 receptors (located on the medium spiny neurones); (iv) the intracellular synthesis of cAMP, which is reduced if the M4 receptors are activated by any available extracellular ACh or agonist drug; (v) the M1 receptors located on the same neurones; (vi) motor side effects resulting from over-stimulation of M1 receptors.

Consider first the situation with standard neuroleptic drugs: Their acute effect is to increase ACh release; and then, as one enters the phase of chronic administration, the D2 receptors proliferate, as do any receptors which are chronically blocked. As a result of this compensatory change, the ability of available extracellular dopamine to reduce ACh release is gradually restored, so that ACh release falls towards, and possibly even reaches its previous baseline level. At the same time, the phase of increased ACh release leads at first to excess stimulation of M1 and M4 receptors. The numbers of both these receptor types then falls over time, as compensation for over-stimulation. (In Parkinson’s disease, where, according to theory, ACh release should also be increased, a similar reduction of M1 receptor numbers has been empirically demonstrated [53]). Eventually, as ACh release falls back towards normal, the numbers of M1 and M4 receptors climb back towards (or even reach) normal levels. In the medium spiny neurones, cAMP formation initially falls and parkinsonian motor symptoms initially appear, but then there is later desensitisation to this effect of increased ACh release. Later still, as ACh falls back to normal levels, resensitisation gradually occurs in the medium spiny neurones, so that the initial decrease in cAMP formation tends to be restored towards (or even reaches) baseline levels, and the initial intensity of motor side effects declines. At the time of drug *withdrawal*, any persisting acceleration of ACh release is abruptly terminated, so that ACh release may fall suddenly, cAMP synthesis in principal neurones abruptly rises, and any remaining motor side effects abate. However, since the number of M4 receptors has returned towards (or even reaches) a normal level, and there is still adequate ACh release, there are still factors limiting production of cAMP. Hence, psychotic rebound, if it occurs is not very florid.

Now consider the alternative scenario, with clozapine withdrawal. It is assumed here that at optimal doses, the effects are exerted mainly at M4 receptors (as agonists) and M1 receptors (as antagonists), but not at D2 or D1 receptors (for which clozapine has lower affinity). Therefore, in Fig. (**1**) the upper two variables (D2 receptor numbers, and ACh release) are assumed to stay unchanged. At the M4 receptors, chronic activation by clozapine will lead to progressive desensitisation (reduction in receptor numbers), with no tendency to normalize, even with prolonged treatment. Production of cAMP, initially sharply reduced will tend to normalize as the receptor numbers drop. The M1 receptors will proliferate, again with no tendency to normalize over time. At the time of *withdrawal* of clozapine, sufficient endogenous ACh may be available to act on the M4 receptors, but its ability to reduce cAMP formation is far below normal, because of the desensitisation of the M4 receptors. The result is that there is little to check production of cAMP, which rises suddenly, dramatically, and beyond the level found with withdrawal of standard drugs. The result is dramatic and florid, a sudden-onset, rebound psychosis, requiring doses of clozapine larger than originally needed before it is brought under control. Even standard neuroleptic drugs will have reduced ability to alleviate psychosis, because their actions depend on adequate sensitivity of M4 receptors. At the same time, due to proliferation of previously-blocked M1 receptors, any typical neuroleptic, used to protect against rebound psychosis, acting by acceleration of ACh release, will produce motor side effects more severe than produced by these drugs prior to the use of clozapine. The striking finding that, on clozapine withdrawal both psychosis and vulnerability to motor side effects are more severe than prior to treatment with this drug indicates that, unlike endogenous ACh, clozapine is an agonist at only one receptor (M4), but an antagonist at the other (M1). However, the receptor changes are transient, and the severe effects of both down-regulation at M4 receptors, and up-regulation of M1 receptors decline over time.

Why are such “rebound” psychotic reactions more severe in previously neuroleptic-responsive patients, than in those who are refractory to such drugs? Probably, because, in the former cases cholinergic interneurones are present in larger numbers. Therefore, during chronic clozapine administration, the M4 receptors are activated not only by clozapine, but also to a significant extent by endogenous ACh. Over the period of chronic administration, the resultant desensitisation of these receptors is then more profound than in refractory patients.

Why are drugs with anticholinergic potency helpful as protection against these rebound effects? This is a difficult question to answer. Antagonists at M1 receptors would be expected to attenuate motor side effects, but antagonists at M4 receptors should release cAMP synthesis from its previous suppression, and therefore should *accentuate,* rather than protect against the “rebound” psychosis. One possible explanation of this may be that some of the commonly-used “anticholinergic” agents (including trihexyphenidyl, benztropine, benperiden, procyclidine, pirenzepine and diphenhydramine), may block actions of ACh at only some of the muscarinic receptors, especially the M1 type, while acting as *agonists* at the critical M4 receptors. Available data [9] show that anticholinergic agents commonly used to reduce motor side effects of antipsychotic drugs have roughly similar affinities for M1 and M4 muscarinic receptors, but there are no data to prove the specific point, that any of these act as antagonists, rather than agonists at M4 receptors. If any such drugs *are* agonists at M4 receptors, they might even have a therapeutic role by themselves.

## 2. ARE THERE ANY OTHER DRUGS IN REGULAR USE, WITH PROPERTIES LIKE CLOZAPINE?

Clozapine is a far from ideal drug. Apart from its psychopharmacology, and the risk of transient rebound reactions on its withdrawal, it has a tendency sometimes to cause agranulocytosis, which, if detected *requires* it to be withdrawn. This is a serious matter, since no other drug has proven efficacy equal to clozapine in neuroleptic-refractory psychotic illnesses. Do any other currently-available drugs have a profile similar to clozapine, which was never properly defined?

There are nine criteria by which such an agent might be recognised, based partly on direct empirical evidence, partly on theory developed in this paper (all discussed as principles, in PART I, and the preceding section of PART II of this paper):

- The agent should be antipsychotic with low incidence of acute motor side effects.
- It need not lead to elevation of blood prolactin levels (an effect attributed to D2 blockade [49]). Failure to meet this criterion does not disprove the thesis that a drug has clozapine-like clinical properties, because affinity for M4 and D2 receptors may be very similar.
- It should be effective in refractory psychosis. When evidence is inconclusive, less rigorous early studies may point to the need for more critical tests.
- Some agents fitting criterion [i] owe this to the combination of dopamine D2 and 5HT2a antagonism, without fulfilling criterion [iii]. When an agent fits criterion [i] without having the “SDA” profile, its status is enhanced as a candidate “clozapine-like” drug.
- The agent should have affinities for dopamine D1 or muscarinic M4 receptors equal to, or higher than that for dopamine D2 receptors.
- If an agent does have high relative affinity for M4 receptors, it should act at such receptors as an agonist or partial agonist, not as an antagonist.
- When used in doses effective for antipsychotic therapy, it should have low (>60%) occupancy of dopamine D2 receptors. A specific upper limit cannot be stated, because different drugs vary in their relatives affinities for D1 or M4 receptors, compared to D2 receptors.
- In advanced Parkinson’s disease, a potential clozapine-like agent should alleviate drug-induced psychosis, without exacerbating parkinsonian symptoms.
- On sudden discontinuation, immediate and severe (but transient) psychotic relapse should be more common than the relapse rate seen with typical neuroleptic drugs, or the better-known “SDA” atypical antipsychotic drugs fulfilling none of criteria [iii] and [v-viii]. This is an important criterion, but a difficult one on which to obtain decisive evidence: Withdrawal even of classical neuroleptic drugs may sometimes lead to rapid psychotic relapse (either due to supersensitivity psychosis, or occasionally, even without this complication). On the other hand, even with an ideal clozapine-like drug, relapse on withdrawal need not be immediate: From the theory developed above, increased cAMP production on withdrawal of an M4 agonist is conditional on activation by some other agent, especially excessive dopamine release, acting at D1 receptors. If dopamine release is not excessive, withdrawal-emergent psychosis may not occur even with clozapine. Carefully controlled studies of matched groups comparing withdrawal syndromes with different drugs are needed to provide firm evidence on this point.

Given this list of criteria, four drugs, apart from clozapine are of interest, dealt with in turn below, and summarized in Table **1**. It is not intended to establish a watertight case that any of these agents *is* a clozapine-like drug, but rather to indicate that, in absence of conclusive evidence, there is enough suggestive evidence to warrant more rigorous testing. Criteria [ii], [iv] and [vii] are ones which suggest clozapine-like properties, if fulfilled, but do not exclude such properties, if they are not met. For instance clozapine itself fails to meet criterion (iv): It does have high 5HT2a/D2 affinity ratio.

*Fluperlapine* was developed by Sandoz, to replace clozapine, after that drug was withdrawn in the 1970s. It has higher affinity for D1 than for D2 receptors, and even higher affinity for M4 receptors (PART I, Table **1**). The data are not clear however, since in a functional assay, its potency as an M4 agonist, in reducing cAMP formation (stimulated by forskolin) was much lower. Like clozapine, it has high relative affinity for 5HT2a receptors [83]. It is an effective anti-psychotic agent with low incidence of motor side effects [28,37,72,129,130], and is also effective against drug-induced psychosis in Parkinson’s disease [62,105]. Its efficacy in refractory psychosis is not proven, but is suggested by one clinical trial [37], whose patient group included ten patients in this class, and for which overall results were “better than previous drugs” in 60% of patients (similar to clozapine). There are no published data on its D2 occupancy at therapeutic doses, nor on the likelihood of rapid-onset withdrawal psychosis for this drug. This agent was not widely marketed, because, like clozapine, it had an associated risk of agranulocytosis.

*Thioridazine* was developed in the late 1950s, and was the subject of many clinical studies in the period 1959-1962. It has affinity for M4 receptors similar to that for D2 receptors (PART I, Table **1**), and a little higher than for D1 receptors. However, the form usually available is a racemic mixture, one of the enantiomers having higher affinity for D1 than for D2 receptors. No data are available on the affinity of the different enantiomers for the M4 receptor. In a functional assay it is an effective inhibitor of forskolin-stimulated cAMP formation, but this effect is not blocked by the non-selective muscarinic antagonist atropine (unlike the effects of clozapine, fluperlapine or olanzapine), so its status as an M4 agonist is uncertain [132]. It is not classed as atypical in terms of its relative affinities for 5HT2a and D2 receptors [83]. Its D2 occupancy in humans, when given in therapeutic doses is said to be similar to that for typical neuroleptic drugs, and not unusually low (as with clozapine). However, this statement is based on only two subjects [34], for whom the dose was quite large (300 and 400 mg/day respectively) and for whom it was unclear how close their dose was to the minimum effective dose. In any case, since its affinities for the D1 and D2 receptors are not very different, and would depend on the proportion of the two optical isomers in the preparation used, expectation of major differences from standard neuroleptic drugs in occupancy at therapeutic doses is uncertain.

Two points suggest that it deserves more serious consideration as an equivalent to clozapine, but safer. *First,* although it has never been rigorously tested in neuroleptic-refractory patients, several older less rigorous studies suggest that it might be effective in such cases: One early study [51] found it to be effective in nine out of 28 cases “who had proved refractory to less radical treatment with other tranquillising drugs”. Another [67] compared it with several classical neuroleptics. In a trial over 24 weeks, the “drop-out” rate due to lack of improvement or deterioration was 12-15% for the drugs chlorpromazine, chlorprothixene, fluphenazine, and triflupromazine, but was only 4.7% for thioridazine (based, for each drug, on ~85 patients). This suggests that thioridazine was effective in some cases refractory to treatment with the other drugs. In another study, good responses were described to thioridazine in seven patients refractory to chlorpromazine, haloperidol, or thiothixene [124]. In a fixed-dose trial of thioridazine in 53 patients [17], all patients eventually improved, and all but three improved quite substantially, being discharged either “markedly improved” (77%) or with “symptoms in remission” (15%). This is a possible indication that it is effective even in refractory patients. However, two studies [43,48] failed to find thioridazine superior to standard drugs in refractory patients. Thioridazine has also been recommended for treatment of drug-induced psychosis in Parkinson’s disease [55]. If it could be proven that thioridazine was effective treatment in refractory psychosis, it would be a viable alternative to clozapine, since the risk of cardiac complications, though higher than for most antipsychotic drugs, is still quite low [99].

The *second* point of possible similarity to clozapine is the pattern of events occurring after sudden withdrawal, in patients receiving thioridazine for some time. The evidence on this is scanty and imprecise. It is certainly true that discontinuation of thioridazine can sometimes be achieved without major problems [1,10,68], and withdrawal-emergent difficulties do not necessarily amount to psychotic exacerbation [63]. Sudden withdrawal of thioridazine is usually initiated by patients, so precise documentation is less likely than for clozapine, where closer scrutiny is now common (due to the risk of agranulocytosis). For sudden-onset psychotic relapse, there are usually more pressing concerns, and so this topic has not been the object of systematic study. In the UK, thioridazine was widely prescribed, especially for intellectually-disabled persons in institutional care, but was withdrawn in 2002, following concern about possible effects on the ECG. When used in intellectually-disabled persons, diagnosis is likely to be inexact, with much heterogeneity within the groups studied. Communication difficulties may also prevent exact recognition of psychotic states on withdrawal. However, the fact of withdrawal-emergent problems is more likely to be documented in such persons. Given these caveats, it is reported that, after long-term thioridazine treatment, behavioural disturbance, or extreme anxiety may occur on discontinuation [7,23,111], sometimes leading to hospitalisation under section [30]. These difficulties include some cases of definite rapid psychotic relapse [68]. Decompensation could not be corrected by standard doses of other neuroleptic drugs [23], a pattern similar to that reported with clozapine (see above). The present author, when much younger, had a serious psychiatric illness, was prescribed thioridazine, which was taken in small doses for nearly forty years (1968-2007; ~300 mg/d for the first two years; but mainly 50 mg/d since then). On three occasions between 1969 and 1980 he tried, very carefully, to withdraw from this medication. On all three attempts he was prevented from this, within days of complete cessation of the medication by resurgence of psychotic symptoms, leading on one occasion (in 1973) to readmission to hospital, an account of which has been published [88]. An enquiry made to the UK Pharmacovigilance service (e-mail address: Pharmacovigilance@mhra.gsi.gov. uk) revealed (26.9.08) 17 reports of adverse reaction on discontinuation of thioridazine in the “CNS/Psychiatry” area. These included individual cases described as: “psychomotor hyperactivity”, “abnormal behaviour”, “aggression”, “agitation”, “anger”, “anxiety”, “hallucinations, visual”, “restlessness”, “intentional self-injury”, “mental status changes”, “suicidal behaviour”, “suicidal ideation” and “suicide attempt”. Most of these descriptions are not precise enough to identify psychotic decompensation, perhaps because they were documented in intellectually-impaired persons. Nevertheless, they do constitute evidence for a syndrome of abnormal behavioural or psychological activation on discontinuation of this drug.

*Mesoridazine *is a metabolite of thioridazine. In addition to the site of asymmetry possessed by the thioridazine molecule, another such site is introduced during its formation. Thus there are four optical isomers to take into consideration. In its racemic form, affinity for M1 and M4 receptors is a little lower than for D2 receptors, but it is not known whether it is an agonist or antagonist. Data for individual isomers on affinity are available for the dopamine D1 and D2 receptors, but not for the muscarinic M1 or M4 receptors (PART 1, Table **1**). It is thus possible that specific enantiomers may have affinity for M1 or M4 receptors equal to or greater than those for dopamine receptors. It is not clear whether it fits the “SDA” profile. Clinically, in its racemic form, two studies show it to be antipsychotic without causing motor side effects [43,124], these two also providing tentative evidence for its efficacy in refractory patients. Nothing is known of its occupancy of D2 dopamine receptors when given in therapeutic doses, its efficacy against drug-induced psychoses in Parkinson’s disease, or the risk of rapid-onset withdrawal-emergent psychosis.

*Quetiapine* is a recently-developed drug, but has properties distinct from most atypical antipsychotics with the “SDA” profile. Its status is rather enigmatic. It probably has low relative affinity for both D1 dopamine and M4 muscarinic receptors compared to dopamine D2 receptors (PART I, Table **1**), and, whatever its affinity, it appears not to act as an agonist at M4 receptors. It has affinity for 5HT2a receptors similar to that for D2 receptors [17,126,134], and therefore could fit the “SDA” profile. Nevertheless, it has some clozapine-like clinical properties, unlike those of other “SDA” atypical antipsychotic agents. In single-case studies of psychosis unresponsive to other treatments (sometimes in combination with other atypicals, or at high-doses), it has been reported to be effective [11,16,96], and in some controlled studies [102] it has shown favourable responses in refractory patients (though not superior to other atypical drugs). However, in three controlled studies, two of them large trials, it was not superior to other atypicals [18,79,116]. It is useful in drug-induced psychoses of Parkinson’s disease [26,35] (using relatively low doses, as also recommended with clozapine), and, for this, is preferred to other atypicals [40]. In group comparisons with clozapine in such patients clozapine is found to be equal [92] or superior [29,60] to quetiapine. Nevertheless, there are hints that different groups of patients are helped by these two drugs: Switch from clozapine to quetiapine may be difficult [35], but in some cases proves valuable when clozapine at maximum tolerated doses is ineffective [54]. It has antipsychotic potency at low levels of D2 occupancy [47,58,65,119]. It is associated with a substantial risk of rapid withdrawal-emergent psychotic rebound [36,73]. In a single case study [126] rapid transfer from clozapine to quetiapine was not accompanied by any rebound, suggesting cross-sensitisation between the two drugs.

Quetiapine is difficult to accommodate within the theory presented in this paper, because some of the criteria derived from that theory (antipsychotic actions at low D2 occupancy, relative lack of motor side effects, effectiveness in drug-induced psychoses of Parkinson’s disease, rapid-onset withdrawal-emergent psychoses, and possibly effectiveness in some refractory psychoses) are met, while other essential criteria (lack of high relative affinity for either the D1 or M4 receptors) are not. It is possible that some of the evidence published so far, or the reasoning behind the theory are incorrect. A more interesting possibility is that there are causes of neuroleptic-refractory psychoses with bases other than those discussed here (i.e. loss of striatal cholinergic interneurones), these causes being rectified by quetiapine but not clozapine. This possibility is made more plausible by the fact that *some* of the patients refractory to standard neuroleptics are also refractory to clozapine. Such patients may yet be responsive to quetiapine. The less-certain basic and clinical evidence about quetiapine needs confirmation before this possibility becomes more definite.

## 3. DEFINITION IN PRACTICE OF INDIVIDUAL SENSITIVITY TO ANTIPSYCHOTIC DRUGS

**Fig. (2):**
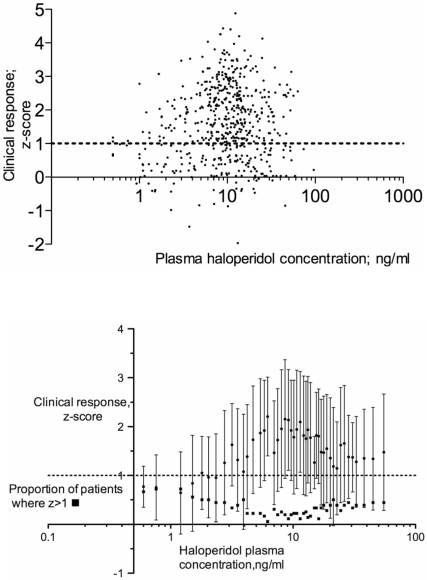
**A (Upper)**: Plot, for 558 individual psychotic patients treated with haloperidol, of clinical response (expressed as z-score, on vertical axis) versus plasma concentration (ng/ml, log scale, on horizontal axis). See Table II for details of studies from which data were obtained. **B (Lower)**: The same data as in A, with patients divided into 46 overlapping subgroups according to ranges of plasma haloperidol levels. For each subgroup, the graph shows mean clinical response (filled circles) ± SD, and the proportion of patients who failed to give greater-than-placebo clinical response (z≥1) (filled squares). (Left-most subgroup: n=9; subgroups with plasma levels between 7 and 14.5 ng/ml: n=36; all other subgroups: n=18; each patient represented in each of two adjacent groups).

Is there any way to define the range of individual sensitivity to antipsychotic drugs in acute treatment, based on therapeutic response, rather than on the subtle motor changes underlying Haase’s neuroleptic threshold? Is there a *practical* way of determining individual sensitivity, to guide dosage used? Any answers to such questions require documentation of individual rather than group-averaged clinical response. It is then better to take plasma concentration rather than dose as the independent variable, this measure being closer to clinical response. A number of papers relate clinical response to plasma level of various antipsychotic drugs, and give values of both variables for individual patients. The drug haloperidol is used in the largest number of such studies, this drug having no active metabolites. For oral haloperidol, the twenty such studies (Table **2**) give data on 558 patients, recently admitted to hospital after acute exacerbations, or studied as chronic in-patients after a placebo “washout” period. The studies use several different clinical rating scales, and some use “difference scores” others using “percent change”. To give equivalence of clinical response measures across studies, the “change” measures for each study are transformed to “z-scores” (change score, as difference or percent, divided by the standard deviation for the change score in that study). (In principle, additional studies using other drugs could be plotted on the same graph, but this would involve the difficult task of matching “clinically equivalent plasma levels” between drugs.) For haloperidol, the resulting plot, with the horizontal axis (plasma level of haloperidol, in ng/ml) on a logarithmic scale is presented in Fig. (**2A**). Fig. (**2B**) presents an analysis of the same data, showing means and SDs of the z-scores (dividing the total number of 558 patients, ranked according to plasma level, into 46 subgroups of 9-36 patients each, for overlapping ranges of plasma levels (see legend to Fig. **2B** for details). Several points are clear from Fig. **2A**:

- There is great spread of measured clinical response at any plasma level, with no sign of bimodality of response (as might be expected if “responders” were separate from “non-responders”).
- Given such wide variability of response, there is also a graded increase with plasma level, in the “maximum possible” response (seen in the “best responders”). This occurs for plasma levels between about 2 ng/ml and 7 ng/ml. Therefore, at least for these “best responders”, an S-shaped dose-response curve appears to fit the relationship with plasma level, with ranges at low and high values of plasma concentration where clinical response does not improve as the plasma level increases, and an intermediate range with progressive increase in clinical response with plasma level increase. At least for these “most sensitive” patients, the clinical response to anti-psychotic drugs appears not to be “all-or-none” (a “step-function” of dose) as suggested by Haase’s “neuroleptic threshold” concept. The relation between plasma level and clinical response is shown more explicitly in Fig. **2B**. Here the fraction of patients who fail to respond is high (~75%) at the lowest plasma levels, and falls between plasma levels of 2 to 7 ng/ml, while at the same time, the extent of average clinical response climbs to z>2. For 246 patients with “optimal” haloperidol plasma levels between 6.5-14.5 ng/ml, only 25.2% of patients failed to respond to an extent z≥1.Fig. (**2B**) gives an indication that the most sensitive patients may begin to show a clinical response for plasma levels as low as 1.5 ng/ml, while the least sensitive ones may not show a response until the level exceeds 7-10 ng/ml. The lower and upper “tails” in the distribution of individual sensitivity are defined by very few patients, so the exact points of maximum and minimum sensitivity are not defined precisely. It is likely that the complete range of individual sensitivity from most to least sensitive patients spans at least a ten-fold range of plasma concentrations, a conclusion in agreement with that reached from estimate of “neuroleptic threshold”, or minimum proven maintenance doses (PART I, Sect. 5).
- In previous attempts to establish a relation between dose or plasma level and clinical response to antipsychotic drugs, there have been hints that, at high doses or plasma levels, the clinical response is less than at moderate doses. In other words, there is a “therapeutic window” for dose or plasma levels [25,44,64,112,125]. This concept has been discussed in earlier publications by the present author [86,87]. In individual studies, the number of data points is too small assess it critically. Fig. (2) permits it to be tested more rigorously. For plasma levels above 14.5 ng/ml, averaged clinical response falls to a mean response (z-score) of 1.4±1.03, a value different from that in the optimal range of plasma levels to a high degree of statistical significance (p>0.0001). The difference between optimal and supra-optimal ranges for plasma concentration has also been found to a statistically significant level within a single study [22]: Patients with acute exacerbations, and haloperidol plasma levels >25ng/ml showed less clinical response, and took longer to reach a criterion levels of improvement than those with lower levels, even though, at such high plasma levels, motor side effects did not differ between groups.

Several recent studies of dose-response relationships for antipsychotic drugs [22,52,69,78] have excluded from the analysis patients who fail to respond beyond a criterion level, even with large doses, and long trial durations. For such patients, the clinical response should not exceed “placebo” effects. How much of the clinical response can be attributed to “placebo effects”? This issue has been discussed in a meta-analysis [127]. Placebo effects (measured using BPRS total scores) ranged from effect sizes of +0.29 (deterioration) to -0.76 (improvement), with a mean of -0.13. The effect tended to be larger (more negative) for short duration trials, perhaps due to non-specific factors stabilizing the patients’ state, as a result of hospitalisation. Specifically, the placebo improvement fell by ~1 BPRS unit per week of the trial. This is a small proportion of the improvement over 4-6 weeks, and is therefore not a major confound for group averages. This paper also gives SDs of change scores for each study, from which, for each study one can compute the 1SD range for individuals within that study. For 17 studies, comparing 810 patients in trials lasting 3-8 weeks, the weighted mean SD for the placebo groups was 14.78 BPRS units. From the 20 studies used to plot Fig. **2**, three [4,70,71] give individual data (in total for 73 subjects) on “BPRS total” change scores, expressed as difference rather than percentage change. From this, the SD for the change score is 13.59 BPRS unit, quite similar to the weighted mean SD for the placebo groups in the meta-analysis of placebo effects [127]. Therefore, in Figs. (**2A**) and (**2B**), a horizontal dashed line is drawn at z=1, to represent the approximate limit of “placebo response”. Responses greater than this are then likely to be drug effects. Using this criterion, the fraction of patients who fail to respond above z=1 rises (Fig. **2B**; square symbols), from 25.5% in the optimal range to 37.58% for plasma levels above 14.5 ng/ml (0.05>p>0.01). If non-responding patients (with z<1) are excluded from the analysis, the clinical response (expressed as a z-score) of the remaining patients is again lower in the high than in the optimal plasma level range (2.04±0.73 *versus* 2.25±0.85), although to a lesser degree of significance (p=0.035) than for the total number of patients in each group.

Several explanations can be offered for the apparent decline in clinical response at high plasma levels. It might be suggested that the decline is an artefact arising because the rating scales used change when high doses are used, for instance because the total symptom picture includes a higher proportion of negative symptomatology. It is also possible that improvement of psychotic symptoms is not seen so clearly in patients with prominent motor side effects. This is compatible with the analysis shown in Fig. **2B**: Using the formula of Fitzgerald and co-workers [38], one can infer that an approximate doubling of plasma levels would be needed to go from the 65% occupancy (said to be the threshold for therapeutic effects) to that producing 80% occupancy (threshold for major motor side effects). In Fig. **2B**, the plasma concentrations over which optimal responses are obtained also extends over a roughly two-fold range. A further explanation of the decline in response at high doses, based on theory similar to that used here, was offered by Miller [85,86]. Just as acquisition of some symptoms, such as delusional beliefs, requires modifiability of beliefs (equivalent at the biological level to dopamine-mediated synaptic change), so also does *recovery* from the same symptoms. At the highest plasma levels and highest D2 receptor occupancy, ACh release may be so high that cAMP formation is reduced so far that such modification is no longer possible. To evaluate this idea requires detailed psychopathological study in the recovery phase, to uncover exactly which symptoms fail to respond at high doses.

**Fig. (3):**
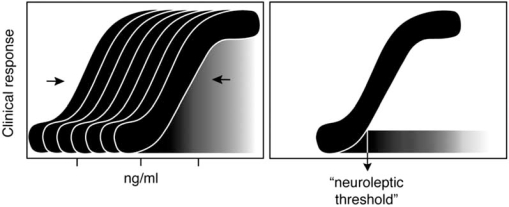
**Diagram to illustrate one possible interpretation of spread of data points in Fig. (2)A.** *Left*: a number of S-shaped individual dose response curves, each “blurred” in the vertical dimension, are displaced to different degrees in horizontal dimension (representing different individual sensitivities to neuroleptic drugs). Right: If all the curves in the left-hand diagram are standardized to the individual “neuroleptic threshold, so that they all overlap, the distinction between “responders” and “non-responders” becomes clearer.

Even after excluding placebo responses, there is great variability in measured clinical response. This is due to several factors: Two of these are: (i) imprecision in the rating scales used (strictly “uncertainty” rather than “variance” in clinical response); (ii) differences between patients in the completeness with which symptoms can be eliminated, due to a variety of individual characteristics, such as personality variables independent of their illness, persisting effects of past episodes etc. These factors would lead to *vertical* spread of individual data on a hypothetical S-shaped dose-response curve. However, bearing in mind the possibility that there is wide individual variation in sensitivity to the drug, the distribution of clinical responses across patients may reflect superimposition of a number of “vertically blurred” S-shaped curves, displaced to varying degrees in the *horizontal* axis (Fig. **3**, left). For the sloping part of each curve, such horizontal displacement would contribute, as a third variable, determining the apparent vertical spread of points. If this were the case, and individual variation in sensitivity is correctly assessed in Haase’s handwriting test, or similar sensitive measures of motor changes, a prediction can be made: If the clinical response in each patient were corrected for differences in neuroleptic threshold, rather than being expressed as a function of plasma levels, all the “blurred” S-shaped curves would be collapsed to the same position in the horizontal axis; and then, the vertical spread of the clinical response measures, after excluding “placebo effects” would be reduced. A bimodal spread of clinical responses in the total population might then appear, with clear separation of “responders” from “non-responders” (Fig. **3**, right).

In practice, tests such as the handwriting method of Haase cannot be used reliably unless they are quantified. Measures of the area of a standardized piece of text written by each patient have been found to correlate across patients with occupancy for dopamine D2 receptors [66]. A more sophisticated quantified version of the handwriting test has been developed [13], based on kinematic analysis of movement velocity, and its scaling with movement amplitude, when subjects write a single standard word. This method has high sensitivity and high ability to distinguish normal subjects from those with idiopathic or drug-induced parkinsonism. Dose-response effects have yet to be published. For the purpose of theory-testing it is preferable to evaluate the test first using pharmacologically-simpler drugs, such as haloperidol. This might then lead to the test being used in clinical practice in the following way: Patients are admitted to hospital in acute psychotic states. In the initial “test” phase of management, lasting no more than one or two days, their disturbed state is checked without using antipsychotic drugs (for instance with benzodiazepines). During this initial phase, small doses of haloperidol are given, and the change in their motor performance is assessed using a quantified version of the handwriting test. As a result, individual sensitivity to antipsychotic drugs is determined. For the therapeutic phase of management of the patient’s illness, this measure is then used to indicate optimal individualized dose of a regular medication (such as an atypical drug), rather than the current “standard” doses based on “group averages”. 

A few studies have been published showing the relationship between clinical efficacy and plasma levels for atypical antipsychotic drugs (risperidone, in maintenance treatment of chronic schizophrenia patients [75], and olanzapine [76]). At present there are insufficient data for such drugs to draw meaningful conclusions from plots such as those shown in Fig. **2**. However, with a sensitive quantitative test [13], motor side effects of atypical drugs may also be quantifiable, and used to guide decisions about individual doses.

A related issue is whether optimal, or minimum-effective doses needed for stable relapse-free maintenance are the same as or smaller than those needed in treatment of acute psychosis. Miller [87] presented a tentative case that maintenance was possible with smaller doses than required in the phase of acute illness. However, the differential was small and the evidence-base quite limited. No studies define the lower limit of plasma levels of any antipsychotic drug compatible with relapse-free maintenance. Thus, empirically the issue is unresolved. Nevertheless, a recent paper [39] provides a theoretical base supporting the differential suggested by Miller [87]. The argument starts from data in undrugged subjects [2], showing that normal occupancy of D2 receptors by dopamine is ~8.8%, compared with ~15.8% in a sample of subjects experiencing psychotic exacerbation (with enhanced dopamine release). If D2-blocking drugs are given to such patients to reduce occupancy by dopamine to normal levels, the required occupancy by the drug is 48%. However, assuming that dopamine and the antipsychotic drug compete for the same receptor sites, and also (as is probable), that excess dopamine release is a *transient* consequence of psychosis, rather than an enduring feature of schizophrenia, calculations based on dopamine occupancy levels during psychosis would overestimate the required occupancy by the drug during maintenance therapy. In maintenance, lower doses would then suffice than in alleviation of acute illness.

## 4. POINTS NOT RESOLVED, AND SUBSIDIARY IMPLICATIONS

If the foregoing theory is correct it requires reinterpretation of some earlier work, and other issues are left unresolved:

- In estimating D2 occupancy by antipsychotic drugs assumptions are made about total number of striatal dopamine D2 receptors (whether or not occupied by drugs) based on averaged data in normal subjects. Drug occupancy in patients is derived from the extent of radio-ligand binding (in the caudate-putamen) in patients, subtracted from that in undrugged controls. The difference is used to calculate binding in patients by the antipsychotic drug. However, if the foregoing theory is correct, the total number of receptors in the striatum will be reduced in patients refractory to, or with low sensitivity to neuroleptic drugs [131]. As a result total available receptors are overestimated, and the fraction of available receptors binding to a ligand is underestimated in such patients. The drug-bound fraction is then overestimated. There are some indications of this in previous studies: (i) It was reported [20] that average occupancy of D2 receptors in 6 refractory patients, receiving typical neuroleptic drugs was 97% (range: 92-100%). The low ligand binding in the patients, from which these values were derived, may have reflected, in part, low receptor numbers rather than high occupancy by the drug. (ii) It is also claimed [21,46] that D2 occupancy is similar in groups of responsive and refractory patients, despite the fact that the latter received larger drug doses the former. Such results may have arisen because the refractory patients had fewer D2 receptors, compensated by the fact that they were more completely occupied than in the other patients. (iii) It was reported that, after discontinuation of a depot neuroleptic drug (haloperidol decanoate), computed D2 receptor occupancy did not fall back rapidly to normal. Even twelve months later it was estimated as 20% [94a]. However, this result, based on reduced ligand binding, might indicate loss of D2 receptors rather than continued occupancy of them by drugs. The method might even allow detection of progressive loss of receptors during prolonged high-dose treatment, although, for ethical reasons, this cannot be done in a planned way.
- In discussion of specific atypical drugs with potential clozapine-like properties, some uncertainties and inconsistencies about receptor affinities remain in available data, and there are uncertainties in their functional neurochemical effects (whether they are agonists, antagonists, or partial agonists). More data are needed on affinities of isomers of mesoridazine and thioridazine, especially for M1 and M4 muscarinic receptors, to clarify whether any of these are safer alternatives to clozapine. More basic data on the receptor-binding profiles and functional effects of quetiapine are also needed, possibly including receptors other than those considered here, to clarify its distinctive mode of action.
- Several interrelated questions remain unanswered about 5HT2a receptors: How do 5HT2a receptors contribute to the low incidence of motor side effects in antipsychotic drugs with the “SDA” profile? To answer that question, more basic questions must first be answered: What is the cytological location of the 5HT2a receptors? What is their action at the cellular or subcellular level?

A single study [101] provides information about the cellular location of 5HT2a receptors in the striatum. They are found predominantly in the dendrites (but rarely in dendritic spines) of cells identified as spiny neurones. Some of these receptors were also located on axon terminals, which, when identified, were similar to excitatory glutamatergic afferents to the striatum. No mention is made of 5HT2a receptors on elements identified as somata or axons of cholinergic interneurones. There appear to be no electrophysiological data from which cellular actions of 5HT2a antagonists in the striatum can be inferred. However, some indirect functional evidence in humans leads one to suspect that 5HT2a antagonists act at a target “downstream” from the D2 dopamine receptors. Thus, motor side effects produced by olanzapine are less than those produced by haloperidol at equivalent D2 occupancy [80,98]. Two studies [61,118] find that, for patients with equal levels of D2 receptor occupancy, motor side effects are usually more severe if the drug used is haloperidol than if it is one of several “SDA” type antipsychotic drugs. However, with risperidone, the severity of side effects was not much less than with haloperidol at equivalent occupancy (see also [66,94b]). In the case of olanzapine, the lack of motor side effects may be due in part to action at muscarinic receptors (PART I, Table **1**). Apart from olanzapine and risperidone, the evidence suggests that other drugs in the “SDA” class owe their relative lack of motor side effects to the fact that consequences of D2 blockade (presumably on the cholinergic interneurones) are reduced by actions “downstream” from this primary target. Arguments presented above suggest that motor side effects arise from the actions of ACh on muscarinic M1 receptors located on medium spiny neurones. This implies that there may be an intracellular site in such neurones where effects produced by M1 and 5HT2a receptors interact synergistically, such that 5HTa antagonists mitigate the effects of stimulation of M1 receptors by endogenous ACh.

Apart from their low tendency to produce acute motor side effects, atypical drugs of the SDA type also have a low risk (compared to classical neuroleptic drugs) of producing the more serious motor side effects, such as tardive dyskinesia, during long term administration [74]. It has been proposed that these more-or-less irreversible conditions are also the result of progressive loss of cholinergic interneurones, as a result of their prolonged overactivity, and associated acute motor side effects [89]. For clozapine the low risk can be understood on the basis of this hypothesis, and the related theory, because the therapeutic action of this drug does not depend on D2 blockade and such prolonged overactivity. However, for other atypical drugs with an uncomplicated “SDA” profile, the therapeutic actions *do* still apparently depend on D2 blockade, and so would be expected to be associated with risk of developing tardive dyskinesia, regardless of the low risk of acute motor side effects. Tardive dyskinesia with these drugs *is* sometimes observed [117], but the risk is lower than with classical neuroleptic drugs. This is paradoxical in the light of the theory presented here: If their therapeutic action depends on blockade of dopamine D2 receptors, and the effect of concomitant 5HT2a antagonism is exerted “downstream” from the cholinergic neurones, these drugs would still be expected to lead to cholinergic cell loss. However, motor side effects, while due primarily to simple blockade of D2 receptors, and overactivity of cholinergic interneurones, may perhaps be intensified by some sort of positive feedback loop, involving neural activity in the medium spiny neurones, and connectional loops through basal ganglia, thalamus and cortex, feeding back to the striatal cholinergic interneurones. Breaking this loop, possibly by the action of 5HT2a antagonists acting on medium spiny neurones, could then reduce the excess activity in the cholinergic neurones, even if such drugs have no direct action on those neurones.

## 5. SUMMARY, SUGGESTIONS FOR PRESCRIBING PRACTICE, AND PREDICTIONS FOR ADVANCEMENT OF SCIENTIFIC UNDERSTANDING OF ANTIPSYCHOTIC DRUGS

Despite the undoubted therapeutic role of antipsychotic drugs, many issues remain unresolved about their mode of action. These include the receptor type(s) which are the ultimate target of these drugs, the reasons for lack of response in some patients with psychotic conditions, the individual variation in sensitivity to their beneficial effects, and the shape of the dose-response curve. The present paper builds upon earlier theoretical work, to address these issues, and argues for the following propositions:

- Although therapeutic potency for most antipsychotic drugs scales with affinity for dopamine D2 receptors, several pieces of evidence do not fit their being the ultimate target. These suggest that D2-blocking drugs act indirectly, with involvement of other receptors, including the dopamine D1 receptors (and others) which can control the mechanisms for synthesis of cyclic AMP.
- Motor side effects of the classical neuroleptic drugs depend on disinhibition of striatal cholinergic interneurones, leading to increased ACh release, with side effects probably depending on actions at muscarinic M1 receptors, located on principal neurones of the striatum. Muscarinic M4 receptors located on the same neurones suppress cAMP formation if this is activated from another source. The net effect of stimulating these receptors is similar to that produced by dopamine D1 antagonists. It is proposed that this is the site at which classical neuroleptic drugs exert their indirect action, leading to their therapeutic benefits.
- It is well established that many patients with psychotic illnesses fail to benefit from classical neuroleptic drugs. Sometimes this feature is present right at the start of the illness, while in other cases it emerges during, and probably as a result of prolonged neuroleptic treatment (“neuroleptic-induced supersensitivity psychosis”). For patients who do respond favourably, there is wide (at least ten-fold) variation of individual optimal or minimum-effective doses. These inter-subject variations do not appear to have a basis in pharmacokinetics, but arise from variation in responsiveness within the brain. The source of uncontrolled variation is “downstream” from the dopamine D2 receptors, at which these drugs exert their direct actions. It is proposed that this unaccounted source of variation is the density of cholinergic interneurones in relevant parts of the striatum, patients becoming more refractory or insensitive to the antipsychotic effects, the lower the density of these neurones. Differences in neuroleptic sensitivity, and density of striatal cholinergic interneurones in different strains of mice support this proposal. Low density of striatal cholinergic interneurones may also predispose patients with Parkinson’s disease to dyskinesias during treatment with dopamine agonists. Tardive dyskinesia arising during long-term neuroleptic treatment probably has a similar pathological basis.
- There is evidence that the drug clozapine has actions at muscarinic M4 receptors as an agonist, with higher affinity than for either D1 or D2 dopamine receptors. This can account for its action in refractory psychosis, where the indirect mode of action via the cholinergic interneurones is no longer available. The effectiveness of this drug against dyskinesia or psychosis in Parkinson’s disease patients also follows from reasoning developed here.
- A further singular feature of clozapine is that rapid-onset severe psychotic rebound is common on discontinuation of the drug. This is to be expected from the mechanism proposed above for its action: Drugs which act directly on a target receptor will cause compensatory changes in receptor number, revealed as withdrawal syndromes on discontinuation. Ones which act indirectly are less likely to produce such an effect.

From the theory developed here the question is raised whether drugs other than clozapine, already in use for many years might have the same favourable profile as clozapine (but without the danger of agranulocytosis, which may necessitate its discontinuation). The drugs for which this possibility is most likely are thioridazine and mesoridazine (or one of their optical isomers). The drug quetiapine fulfils several of the criteria for a clozapine-like drug, but, from presently-available data, does not have high relative affinity for either the D1 or M4 receptors. Its status is enigmatic, but might indicate a usefulness in cases of refractory psychosis for which even clozapine is ineffective. In any case, because of the risk of rapid-onset withdrawal psychosis, clozapine or any other drugs in the same class, should not be the first-line treatment for psychosis, but should be reserved only for proven cases of non-responsiveness to standard drugs.

Several predictions follow from the theory presented here:

- It is expected that dopamine-mediated synaptic change in the striatum is partly dependent on reduced cholinergic activation of muscarinic M4 receptors, located on medium spiny neurones.
- The presence of tardive dyskinesia, supersensitivity psychosis (or other sorts of refractory psychosis), and (in Parkinson’s disease) L-DOPA-induced dyskinesias, should correlate with low density of striatal cholinergic interneurones, and cholinergic markers. It may be possible to develop scanning methods for quantifying such markers, to test this prediction in vivo.
- In rodent strains which show catalepsy without a pharmacological trigger, there may be an unusually large number of striatal cholinergic interneurones. Those with a small number of such neurones may have a low threshold for “stereotypy” induced by stimulant drugs.
- More binding data are needed for some of the drugs with suspected clozapine-like pharmacological profiles, and also more data are needed to define whether some drugs acting at M1 and M4 muscarinic receptors are agonists, partial agonists, or antagonists.
- The relation between plasma levels of antipsychotic drugs and clinical responses show a wide spread in the response measures at any effective plasma level. The “neuroleptic threshold” concept of Haase suggests that the dose producing the least detectable motor side effects (for typical neuroleptic drugs) is also the dose producing all, or most of the therapeutic benefit, although in this paper it is argued that this is not a sharp all-or-none effect. It is suggested that the clinical response might become distinctly bimodal (corresponding to responder versus non-responder status), if responses are standardised with respect to individual neuroleptic threshold (rather dose or plasma level). A method is suggested by which quantitative determination of the individual threshold might be used to determine individualized optimal doses for the modern generation of antipsychotic drugs.
- Suggestions are made about methodological errors in estimation of drug occupancy of dopamine D2 receptors when these estimates are made in neuroleptic-refractory or insensitive patients. The possible reasons why atypical drugs of the “serotonin-dopamine antagonist” class (other than clozapine) have a low incidence of motor side effects are also discussed.

## Figures and Tables

**Fig. (1):**
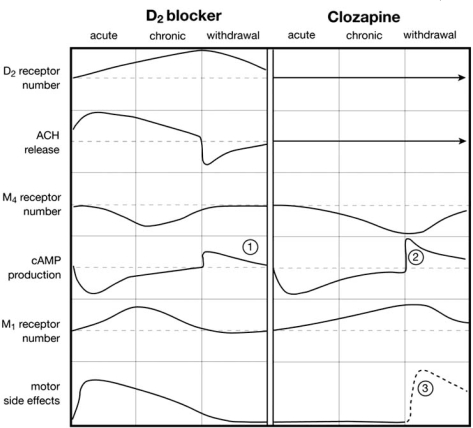
**Events prior to and on discontinuation of chronic regimes of classical (D2-blocking) antipsychotic drugs, and clozapine.** See text. 1: On withdrawal of chronic regimes of D2-blocking drugs, release of cAMP production, and psychotic rebound, if it occurs, is not severe. 2: On withdrawal of clozapine, cAMP production increases with little to hold it back (in the short term), and psychotic rebound may be severe. 3: Likewise, on withdrawal of clozapine, substitution by D2-blocking drugs (dashed trace) may produce motor side effects more severe than such drugs would produce prior to clozapine treatment.

**Table 1.** Potential Clozapine-like Drugs: Synopsis

Drug Criterion	Clozapine	Fluperlapine	Thioridazineor One of its Isomers	Mesoridazine or One of its Isomers	Quetiapine
Antipsychotic without motor side effects	✔	✔[a]	✔	✔[b]	✔
No elevation of blood prolactin	✔	✔[c]	X[c]	?	✔[d]
Effective in refractory psychosis	✔	?(✓)[e]	?(✓)[f]	?(✓)[b]	?(✓)[g]
Fits ‘SDA’ profile	Yes	Yes [h]	No [h]	?	?Yes[i]
High relative M4 affinity	✔	✔[j]	✔[j]	?✓[j]	X[j]
M4 agonist	✔	?✓[j]	?[see text]	?	X[j]
Antipsychotic with low D2 occupancy	✔	?	?	?	✔[Sect 3]
Effective in Parkinson’s L-DOPA psychosis	✔	✔ [k]	?✔[l]	?	✔[m]
Risk of rapid rebound psychosis	✔	?	?✓[n]	?	✔[o]

Notes:

✔ criterion definitely fulfilled;

X: criterion not fulfilled;

?(✓): suggestive evidence that criterion is fulfilled.

Sources:

a: [28,37,72,129,130];

b: [43,124];

c: [32];

d: [19,49,56];

e: [37];

f: [17,51,67,124];

g: [18,79,116];

h: [83];

i: [12,100,134];

j: see Table 1 (PART I)

k: [62,105]:

l: [55];

m: [26,29,35,40,54,60,92];

n: [7,23,30,68,111];

o: [36].

**Table 2.** Studies Giving Individual Data on Plasma Levels and Clinical Response, for Haloperidol in Treatment of Acute Psychosis

Authors, Chronological Order	Trial Duration	Number of Subjects	Patients	Clinical Scale, and Measure	Comments
a	3-12 wk	17	Acute exac	BPRS total improvement difference score	16 out-patients, 1 in-patient
b	4 wk	16	Acute exac	BPRS total improvement difference score	
c	14 d	14	Acute exac	NHSI; % improvement	
d	28 d	10	Acute exac	AMS Global improvement difference score	
e	2 wk	14	Acute exac	NHSI; % improvement	
f	20 d	36	Acute exac	BPRS total improvement difference score	18: manic psychosis 18 schizophrenic psychosis
g	24 d; 1-3 wk washout	27	Chronic in-patients	BPRS-psychosis; % improvement	*Included:* those showing exac during washout; *Excluded:* those known as neuroleptic-refractory
h	42 d	20	Acute exac	BPRS total improvement difference score	
i	42 d; 6 wk washout	19	Chronicin-patients	BPRS-psychosis; % improvement	
j	42 d; > 1 wk washout	44	Chronic in-patient	CGI Global improvement difference score	
k	28 d	13	Acute exac	BPRS-psychosis; % improvement	
l	29 d	16	Acute exac	BPRS-psychosis; % improvement	
m	6 wk; >4 wk washout	30	Chronic in-patients	BPRS-psychosis; % improvement	
n	21 d	28	Acute exac	BPRS-psychosis; % improvement	None known to be neuroleptic refractory
o	2 wk	29	Acute exac	BPRS-psychosis; % improvement	
p	28 d	26	Acute exac	BPRS-psychosis; % improvement	33% of subj dropped out by 28d; no evidence that this biased the results
q	21 d	20	Acute exac	BPRS-psychosis; % improvement	
r	3 wk; 1 wk washout	54	Acute exac	BPRS-psychosis; % improvement	
s	28 d	68	Acute exac	BPRS psychosis improvement difference score	
t	21 d	57	Acute exac	BPRS-psychosis; % improvement	

References:

a: [71];

b: [33];

c: [77];

d: [128];

e: [45];

f: [4];

g: [110];

h: [70];

i: [6];

j: [97];

k: [109];

l: [93];

m: [59];

n: [103];

o: [57];

p: [114];

q: [95];

r: [125];

s: [121];

t: [120].
